# Discharge instructions for parents in the context of pediatric emergency care: a narrative review

**DOI:** 10.1186/1472-6963-14-S2-P20

**Published:** 2014-07-07

**Authors:** Janet Curran

**Affiliations:** 1 School of Nursing, Dalhousie University, Halifax, Nova Scotia, Canada

## Background

Emergency departments are the leading providers of unscheduled care with over 85% of patients discharged home after their visit. Discharge communication with parents has been shown to vary across setting and illness presentation. Emergency practice environments are chaotic by nature and characterized by multiple interruptions. The primary goal of this synthesis project was to understand how and why discharge instructions worked under different conditions.

## Materials and methods

We conducted a narrative review of the policy and empirical literature to examine how and why discharge instructions worked under different conditions in the context of pediatric emergency care. Three stakeholder groups (practitioners, administrators and parents) were actively engaged with the research team during each step of the synthesis process. We collected data on the types of interventions and implementation strategies used in the included studies. We developed a preliminary synthesis using textual descriptions, tabulation and content analysis. We used a taxonomy of behaviour change techniques as a guiding framework to link behaviour change techniques described in the interventions with the relevant theory.

## Results

Email survey of 15 Canadian tertiary care centres failed to identify any existing policies guiding discharge communication in pediatric emergency departments. Following duplicate screening of 4690 abstracts from EMBASE, PubMed and CINAHL and hand searching of 5 major journals, we included 68 studies in the final synthesis. Less than half of included studies (n=30) involved experimental or quasi experimental designs. The majority of interventions were educational strategies targeting parents of children with different illness presentations. Most studies were also carried out in larger urban paediatric emergency departments. The authors identified a range of factors influencing implementation of the interventions including duration of the educational intervention, timing of delivery in the emergency department visit, the mode of delivery, and illness acuity of the child.

## Conclusions

Improving discharge communication for parents and children in an emergency practice setting presents a significant opportunity for increasing adherence to treatment plans and improving health outcomes for children. Knowledge users agree there is an urgent need to address this important policy and practice gap. To date the majority of strategies to improve discharge communication have been educational strategies targeting parents. However, findings from this review have identified a number of barriers that would suggest the need for investigating other types of intervention strategies.

